# Intratumor microbiome in cancer progression: current developments, challenges and future trends

**DOI:** 10.1186/s40364-022-00381-5

**Published:** 2022-05-31

**Authors:** Jinyan Liu, Yi Zhang

**Affiliations:** 1 Biotherapy Center and Cancer Center, the First Affiliated Hospital of Zhengzhou University, Zhengzhou, Henan China; 2 State Key Laboratory of Esophageal Cancer Prevention & Treatment, Zhengzhou, Henan China

**Keywords:** Intratumor microbiome, Immune system, Anticancer treatment

## Abstract

Cancer is a complicated disease attributed to multifactorial changes, which causes difficulties with treatment strategies. Various factors have been regarded as the main contributors, and infectious etiological factors have recently attracted interest. Several microbiomes contribute to carcinogenesis, cancer progression, and modulating cancer treatment by inducing cancerous epithelial cells and chronic inflammation. Most of our knowledge on the role of microbiota in tumor oncogenesis and clinical efficiency is associated with the intestinal microbiome. However, compelling evidence has also confirmed the contribution of the intratumor microbiome in cancer. Indeed, the findings of clinical tumor samples, animal models, and studies in vitro have revealed that many intratumor microbiomes promote tumorigenesis and immune evasion. In addition, the intratumor microbiome participates in regulating the immune response and even affects the outcomes of cancer treatment. This review summarizes the interplay between the intratumor microbiota and cancer, focusing on the contribution and mechanism of intratumor microbiota in cancer initiation, progression, and potential applications to cancer therapy.

## Introduction

The human body comprises a mixture of mammalian and microbial cells, with the latter exceeding the former by nearly tenfold. The microbial genetic repertoire is approximately 100-fold more abundant than that of the human host [1]. Beyond bacteria, the human commensal microbiome consists of viruses, archaea, fungi, and other eukaryotic species [2]. Commensal microbes inhabit at all mucosal barrier surfaces, with the distal gastrointestinal (GI) tract residing the most abundant population [3]. The commensal microbiome is physiologically beneficial to the human host, but perturbed microbiota components or a disrupted mucosal environment could drive immune pathology and systemic inflammation [4] that affects human health. Microbiome dysbiosis contributes to the development of enteritis, pneumonia, and cancer [5, 6].

Cancer is a threat to human health worldwide owing to the high morbidity and mortality rates. All cancer cells are characterized by common hallmarks, including transformation, unrestricted growth, and progression [7–10]. Various factors have been identified that contribute to cancer initiation and progression, including gene mutations, suppressed immune responses, and a complex tumor microenvironment (TME) [11–14]. The tumorigenic and immunomodulatory roles of abnormal microbiomes are now recognized. The existence of the microbiome in tumor sites has been widely validated and accepted [15], and their effects on oncogenesis and progression have been extensively studied [1, 2, 16]. The interplay between the commensal microbiome and clinical treatment efficacy has also been proposed [2, 17]. The intimate interconnection between cancer and microbiota was documented as early as 1550 BCE when tumors were treated by incisions and poultices [18]. However, early attempts to apply microbiota to cancer treatment failed [18–20]. A limited mechanistic foundation might explain this, as technology that could detect low microbiome biomass was restricted. Current research into microbiota and cancer is supported by methods and technologies such as immunohistochemistry, quantitative PCR, immunofluorescence, fluorescence in situ hybridization, electron microscopy, and 16S rRNA sequencing [15].

The contribution of gut microbiota in cancer initiation, progression, and drug resistance has been thoroughly investigated. The gut microbiota can affect responses to chemo- and immunotherapeutic agents by modulating their efficacy or toxicity [21–25]. Therapeutic interventions to modulate microbiota composition to improve immunotherapy efficacy in mouse models have been promising [17, 26–28]. Subsequent endeavors have also translated preclinical findings into early-stage clinical tests with encouraging outcomes [29–31]. Apart from the gut microbiota, the existence and functional importance of intratumor microbiota in cancer remain contentious [15]. This review summarizes the roles of the intratumor microbiota in the tumor microenvironment, responses to therapies, and potential strategies that might facilitate better outcomes of cancer treatment.

### Intratumor microbiome

**Fig. 1:**
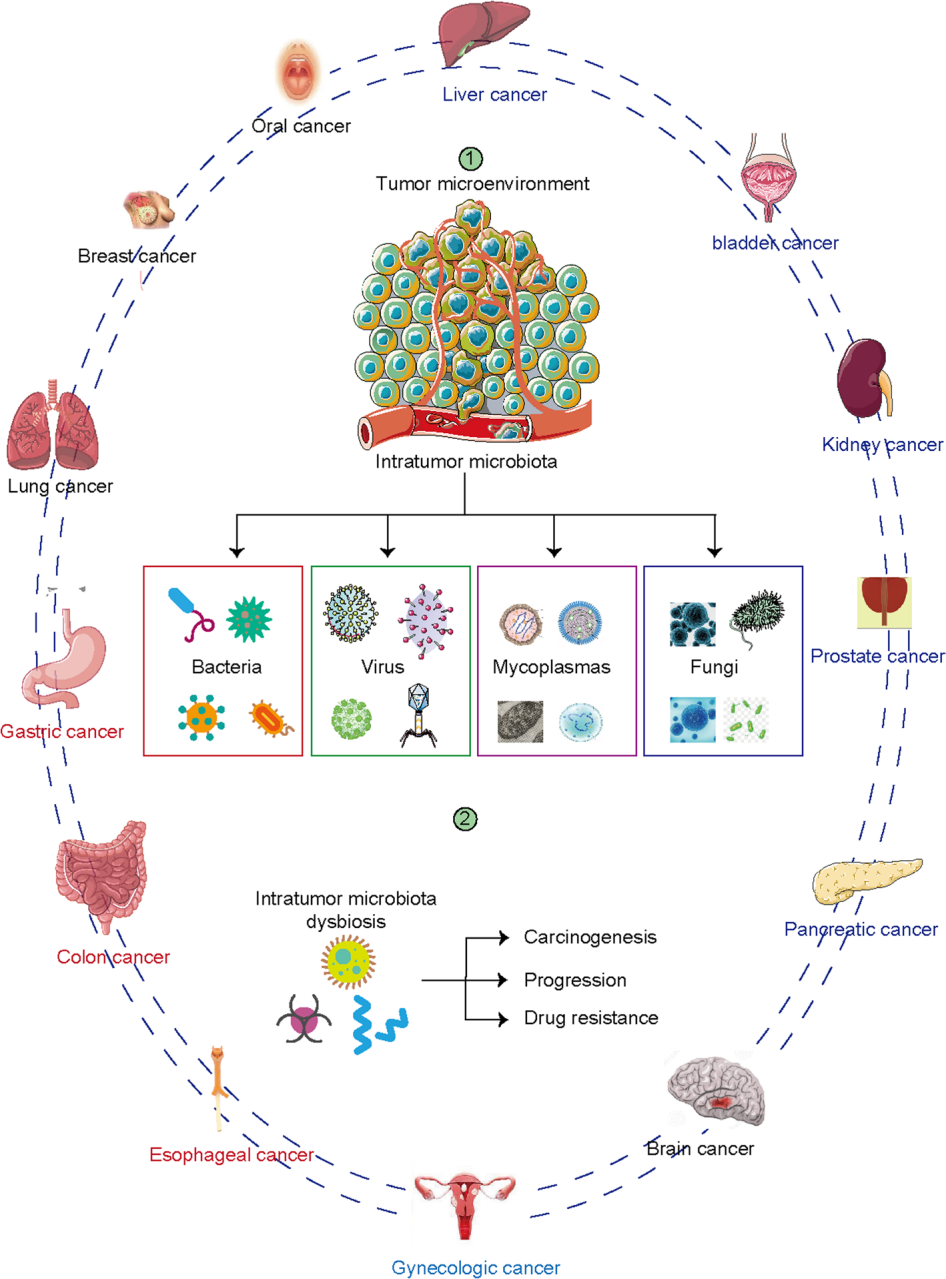
Intratumor microbiota niches across cancer types. Microbiota are detected in multiple solid tumors, including liver, bladder, kidney, prostate, pancreatic, brain, esophageal, colon, gastric, lung, breast, oral and gynecologic cancers. The intratumor microbiome has been convinced to contribute to the carcinogenesis, cancer progression and drug resistance

The intratumor microbiota has received less attention than the gut microbiome. In contrast to intestinal cancer, little is known about correlations between intratumor microbiota and other cancers. However, despite the paucity of studies, the composition of the intratumor microbiota is associated with many types of cancer. Organs and tissues, including the esophagus, lung, breast, prostate, bladder, stomach, kidney, liver, and pancreas, were previously considered sterile. However, next-generation sequencing (NGS) revealed that these organs harbor low-biomass microbial populations [15, 32, 33]. The intratumor microbiome is a major constituent of the tumor microenvironment that affects tumorigenesis, disease progression, drug resistance, and prognosis [34] (Fig. 1).

#### Intratumor tumorigenic bacteria

The human microbiome comprises bacteria, fungi, viruses, and mycoplasmas [35]. Epidemiological, basic, and clinical findings have established a link between intratumor bacteria and increased risk of cancers, suggesting that intratumor bacteria are high-risk factors for many cancers, including oral, lung, pancreatic, prostate, esophageal, bladder, colon, and gastric cancers [15, 36] (Table 1).

**Table 1:** Summary of intratumor bacteria in cancerous tissues and their roles in oncogenesis, progression, and prognosis in cancers

Genus	Status	Cancer	Samples	Role	Mechanism	Refs
Bacteria						
*Thermus* and *Ralstonia*	Dysbiosis	Lung	Adenocarcinoma and squamous cell carcinoma	Cancer histology	NT	
*Legionella*	Enriched		Primary and metastatic lung tumor tissues	Oncogenesis metastasis	NT	[37]
*Acidovorax*	Enriched		Lung cancer tissues with or without TP53 mutation	Linked with TP53 mutation		[38]
*Staphylococcus*	Decreased		Tissues from lung cancer patients with unilateral lobar masses and healthy controls			[39]
*Anaerococcus, Caulobacter, Streptococcus,* and *Propionibacterium*	Decreased	Breast	Tissues from Breast cancer patients, predisposed to breast cancer, and healthy controls	Negatively correlated with oncogenic immune features; positively associated with T-cell activation-related genes		[40]
*Bacteroidetes* and Firmicutes	Lower ratio		Benign and breast cancer			[41]
*Fusobacterium nucleatum*	Enriched		Benign and breast cancer	Poor prognosis	Oncogenesis and suppressed immune response	[42]
*Bacteroides fragilis*	Enriched			Cancer progression	BFT drives epithelial hyperplasia in the mammary gland	[43]
*Lactobacillus fermentum*	Enriched	Esophageal	Tissues from esophageal cancer patients and healthy controls	Cancer screen	NT	[44]
*Campylobacter* species	Enriched		Esophageal adenocarcinoma and control tissues	Prognosis	NT	[45]
*F. nucleatum*	Enriched		Esophageal cancer and normal tissues	Prognosis	NT	[46]
Bacteroidetes/Fusobacteria/Spirochaetes	Decreased		Esophageal cancer and normal tissues		NT	[47]
Actinobacteria	Enriched		Esophageal cancer and normal tissues		NT	[48]
*P. gingivalis*	Enriched		Esophageal tissues from ESCC patients and normal controls	Progression and prognosis	NT	[49]
*Fusobacterium nucleatum*	Enriched		Resected ESCC samples	Chemoresistance	NT	[50]
*Fusobacterium and less Streptococcus*	Dysbiosis		ESCC tumor tissues and normal tissues	Oncogenesis	NT	[51]
*H. pylori*	Enriched	Gastric	Gastric cancer and normal tissues	Oncogenesis	Promote p53 degradation and immune evasion	[52–55]
*E.coli*, butyrate-producing bacterium *SM4/1*, *Oscillatoria*	Enriched	Bladder	Tumor samples with muscle invasive bladder carcinoma (*n* = 400)	Poor prognosis	Positively correlates with EMT-associated genes	[56]
*Staphylococcaceae*	Enriched	Prostate	prostatic tumor, peritumor and nontumor tissues	Oncogenesis		[57]
*Pseudomonas*, *Escherichia*, *Acinetobacter*, *Propionibacterium spp.*	Enriched		frozen radical prostate samples from tumor and adjacent benign tissue	Oncogenesis		[58]
	Enriched		*Propionibacterium acnes spp*	Prostate tissue inflammation		[59]
Proteobacteria	Enriched		Prostatic tumor tissues	Oncogenesis		[60]
Enterobacteriaceae Pseudomonadaceae	Enriched	Pancreatic	Pancreatic ductal adenocarcinoma tissues and normal human pancreas	Chemotherapy resistance	Metabolize chemotherapy drugs	[32]
Proteobacteria Bacteroidetes Firmicutes	Enriched		Pancreatic cancer and normal tissues	Tumor progression	Modulating M1 macrophage/Th1 differentiation, that affect CD8 + T cell function	[61]
*Pseudoxanthomonas Streptomyces Saccharopolyspora Bacillus clausii*	Enriched		pancreatic adenocarcinoma (PDAC) patients with short-term survival (STS, < 5 years) and long-term survival (LTS, > 5 years)	Prognosis	Elevated infiltration and activation of CD8 T cells	[62]
*F. nucleatum*	Enriched	Mouth	Oral squamous cell carcinoma and normal oral tissues	Predictor	Promotes EMT transition	[63]
*Abundance of* Firmicutes *(especially Streptococcus) and* Actinobacteria *(especially Rothia)*	Bacterial dysbiosis		Oral cancers and anatomically matched contralateral normal tissue		Promote oncogenesis and progression	[64]
*Fusobacterium/Prevotella*	Enriched		Oral squamous cell carcinoma tissues and adjacent non-tumor mucosa 5 cm distant	Oncogenesis	NT	[65]
*Peptostreptococcus*	Enriched		Tumor samples from patients with OSCC	Better prognosis	NT	[66]
*F. nucleatum*	Enriched	Colon	Colorectal cancer and paired normal tissues	Oncogenesis and progression	Activates β-catenin signaling; Lower density of CD3^+^ T cells; Recruits immuno- suppressive cells; Inactivation of NK and T cells	[67–73]
*Escherichia coli and Bacteroides fragilis*	Enriched		Familial adenomatous polyposis samples and healthy controls	Promotes cancer initiation	that secrete oncotoxins	[34]
*Fusobacterium*	Enriched		Paired primary colorectal and metastatic tumors	Cancer metastasis	NT	[70]
*Helicobacter spp*	Enriched	Bile duct	Bile duct cancer tissues	Oncogenesis	NT	[74]
*Fusobacterium nucleatum, Escherichia coli, and Enterobacter sp.*	Enriched	Gallbladder	Bile samples from patients with gallbladder cancer and cholelithiasis	Oncogenesis	NT	[75]
Decreased *Nesterenkonia*, and increased *Methylophilaceae*, *Fusobacterium*, *Prevotella*, *Actinomyces*, *Novosphingobium,* and *H. pylori*	Bacteria dysbiosis	Extrahepatic cholangiocarcinoma	Tissues from extrahepatic cholangiocarcinoma (ECCA) and benign biliary pathology (BBP) cohorts	Oncogenesis	NT	[76]
*Helicobacter bilis*	Enriched	Extrahepatic cholangiocarcinoma	Tissues from extrahepatic cholangiocarcinoma (ECCA) and benign biliary pathology (BBP) cohorts	Oncogenesis	NT	[77]
Bifidobacteriaceae Enterobacteriaceae Enterococcaceae	Enriched	Cholangiocarcinoma	primary CCA tumors and matched normal tissues	Oncogenesis	NT	[78]
*Helicobacter species*	Enriched	Hepatocellular carcinoma	Liver samples from patients with hepatocellular carcinoma, non-cirrhotic chronic hepatitis C, and healthy controls	Oncogenesis	NT	[79]
*Sneathia and Lactobacillus*	Dysbiosis	Cervix				[80]
*Fusobacterium spp*	Enriched		Samples from patients with squamous intraepithelial lesions (SIL) and cervical cancer	Oncogenesis	NT	[81]
*L. gasseri*	Enriched			Oncogenesis	NT	[82]
*Atopobium, Porphyromonas, Dialister, Peptoniphilus, Ruminococcus, Anaerotruncus, Anaerostipes, Treponema, Bacteroides and Arthrospira*	Enriched	Endometrium	Uterine samples from cancer and benign disease	Oncogenesis	Modulating the vaginal pH	[83]
*Brucella, Mycoplasma, Chlamydia spp.*	Enriched	Ovary	Ovarian tumor	Oncogenesis		[84]
Proteobacteria	Enriched		Ovarian cancer tissues and normal distal fallopian tube tissues	Cancer initiation and progression	Modulating immune response	[85]
*Actinomyces and Parvimonas*	Dysbiosis	Head and neck squamous cell carcinomas (OSCC)	Paired normal and tumor resected OSCC specimens	Tumor stage	NT	[86]

#### Gastrointestinal cancer

Among all tumors, gastrointestinal malignancies have received the most attention because of the abundance of bacterial residues in the gut [3]. Gut bacteria dysbiosis occurs in patients with adenoma or colorectal cancer (CRC), as populations of *F. nucleatum* [67–69], *Escherichia coli*, *B. fragilis* [34] and *Fusobacterium* [70] are increased, whereas those of *Ruminococcus*, *Bifidobacterium*, and *Streptococcus species* are decreased [87]. Intratumor bacterial dysbiosis is causally correlated with the oncogenesis of CRC [34, 63, 79, 88], metastasis [70], immune evasion [71–73], and drug resistance [89, 90]. The interplay between intratumor bacteria and liver/biliary tract cancers has been extensively investigated because the liver and biliary tract are exposed to the gastrointestinal microbiome through the gut–liver axis. *Bifidobacteriaceae, Enterobacteriaceae*, and *Enterococcaceae* are enriched in tumor samples from patients with cholangiocarcinoma [78]. The population of *Nesterenkonia* is decreased, whereas those of *Helicobacter bilis*, *Fusobacterium*, *Methylophilaceae*, *Prevotella*, *Novosphingobium*, *Actinomyces*, and *H. pylori* are increased in patients with extrahepatic cholangiocarcinoma (ECCA), compared to those with benign biliary pathology (BBP) [76, 77]. The abundance *of Helicobacter species* is high in hepatocellular carcinoma tissues [79].

Abnormal bacterial abundance such as increased *F. nucleatum* [63] and *Fusobacterium/Prevotella* [65] and decreased *Streptococcus/Rothia* [64] in the oral cavity might be a risk factor for oral cancer. Bacterial dysbiosis also correlates with the prognosis of patients [66]. Intratumor bacteria have also been detected in other gastrointestinal cancers, and dysbiosis causally correlates with carcinogenesis, progression, suppressed immune response, and drug resistance. *Helicobacter pylori* was one of the first bacterial species to be directly associated with the oncogenesis of gastric cancer [52–55]. Bacterial dysbiosis [47–49, 51] promotes chemoresistance in esophageal cancer [50]. The bacterial biomass in esophageal tissues can distinguish cancer cohorts [45], cancer types [44], tumor stage, and prognosis [46]. In addition, *Proteobacteria*, *Bacteroidetes*, and *Firmicutes* are abundant in tissues from mouse models and humans with pancreatic cancer [61]. The presence and dysbiosis of bacteria in pancreatic cancer contribute to oncogenesis, immune evasion, resistance to chemotherapy [32, 61] and even affects patient prognosis [62]. Dysbiosis also occurs in cancers of other parts, such as the bile duct [74] and gallbladder [75].

### Genitourinary cancers

Intratumor bacteria participate in the development of cancer in genitourinary organs that were previously considered sterile [15]. Bacterial biomass has been recognized in tissues [59] as well as in frozen samples of prostate tumors and adjacent benign tissues after radical prostatectomy. Over 40 unique bacterial genera have been identified [58] in freshly resected prostate tissues. The abundance of *Staphylococcaceae* [57, 60] and *Propionibacterium acnes spp.* [59] is increased, and the biomass of *Actinobacteria*, *Firmicutes*, *Proteobacteria*, *Lactobacillales*, and *Streptococcaceae* is decreased [57, 60]. Intratumor bacteria with increased *E. coli*, the butyrate-producing bacterium *SM4/1*, and *Oscillatoria* indicate a poor prognosis of bladder cancer [56].

### Gynecological cancers

Microbiota in the lower female reproductive tract protects the endometrium, ovary, and fallopian tubes from pathogen attack and sustains homeostasis [91]. However, bacterial dysbiosis contributes to gynecological malignancies. An increased abundance of *Atopobium, Dialister, Porphyromonas, Peptoniphilus, Anaerotruncus, Ruminococcus, Anaerostipes, Treponema, Bacteroides,* and *Arthrospira* promotes the carcinogenesis of endometrial cancer [83]. Cervicovaginal microbiome dysbiosis correlates with a high risk of ovarian cancer [92]. The prevalence of *Brucella, Mycoplasma,* and *Chlamydia spp.* has been confirmed in samples from patients with ovarian cancer [84]. Both diversity and richness are lower in ovarian cancer tissues than in normal distal fallopian tube samples, with a decrease in Proteobacteria abundance [85]. Bacterial dysbiosis with increased abundance of *Sneathia*, *Lactobacillus gasseri* [80, 82] and *Fusobacterium spp.* [81], and decreased *Lactobacillus* biomass [80] promotes the oncogenesis of cervical cancer, which is the most prevalent malignancy associated with human papillomavirus (HPV).

### Other cancers

In addition to being identified in tumors that arise from mucosal organs, intratumor bacteria have also have been identified in lung, breast, bone, melanoma cancers, glioblastoma multiforme (GBM), and head and neck squamous cell carcinomas [15, 37–41, 86]. Other bacteria, such as *B. fragilis* [43] and *F. nucleatum* [42], also contribute to breast cancer progression. Intratumor bacterial dysbiosis correlates with a high risk of lung cancer in clinical samples [37–39]. Moreover, bacterial dysbiosis correlates with TP53 mutations [38], cancer metastasis [37], and cancer histology [37]. In addition to lung cancer tissues, bacterial profiles differ in saliva, sputum, bronchoscopic samples, and bronchoalveolar lavage fluid (BALF) between patients with lung cancer and healthy controls [37, 39, 93] (Table 1). However, associations between intratumor bacteria and other types of cancers have not been extensively investigated. The profiles of intratumor bacteria in different types of cancer are distinct, with abundance and diversity being the highest in breast tumors [15].

### Intratumor non-bacterial microbiome

Mycoplasma, fungi, archaea, protists, and viruses are also microbiome components [94]. Investigations into the roles of microbes in cancers have mainly focused on bacteria [95]. However, other types of microbes, such as mycoplasmas, fungi, and viruses, also play roles in cancer [96, 97].

### Intratumor mycoplasmas

The interplay between mycoplasmas and malignancy was discovered during the 1960s [97]. Mycoplasma infection is prevalent in colon carcinoma and gastric, esophageal, lung, breast, prostate, ovarian, cervical, kidney, pancreatic cancers, and glioma [84, 98–102]. A direct comparison of samples from patients with small-cell lung carcinoma and healthy controls found significant mycoplasma accumulation in cancer tissues [103]. Furthermore, mycoplasma infection induces transformation and tumorigenicity in the normal human lung cell line, BEAS-2B, and promotes lung cancer angiogenesis by elevating bone morphogenetic protein 2 (BMP2) levels [16]. Mycoplasma infection induces the malignant transformation of other human cell lines, such as A549 (lung) [16], benign prostate hyperplasia (BPH)-1 [36], blood cells [104], SK-UT-1B (uterus) [105], and BE-M17 (neurons) [106]. Although mycoplasmas have malignant potential and are prevalent in various types of cancer, their pathological role in tumorigenesis remains controversial. Besides oncogenesis, mycoplasma infection contributes to drug resistance [32].

### Intratumor fungi

Fungi correlate with cancer risk [96]. Fungi are approximately 3,000-fold more abundant in pancreatic ductal adenocarcinoma (PDA) than in normal pancreatic tissues from mice model and human samples, and most of them comprise *Malassezia spp*. This fungus accelerates oncogenesis in mouse models of PDA, and ablating it represses tumor growth and progression [96]. Mechanistically, *Malassezia* binds through its surface glycans to mannose-binding lectin (MBL) to activate the complement cascade, resulting in oncogenic progression [96]. A fluorescence-tagged fungal strain introduced into the gut of a mouse model that was detectable in the pancreas after 30 min, suggesting that intratumor fungi are translocated from the intestine [107]. *Candida* infection is causally linked to cancer risk. Several putative mechanisms might explain their contribution to oncogenesis. *Candida* produces nitrosamines that alter cell proliferation [108] and secretes cytokines such as tumor necrosis factor-alpha (TNF-α) and interleukin (IL) 18 (IL-18) that modulate the immune response and promote tumor cell adhesion to epithelial cells [109]. Fungi have also been detected in prostate, esophageal, gastric, skin, oral, lung, and colon cancers [60, 107, 108]. However, the underlying mechanisms remain unclear and await further investigation.

### Intratumor viruses

As in the case of other microbiomes, virome infection closely correlates with cancer in solid tumors of colon, hepatocellular, oral, breast, cervical, esophageal, gastric, and lung cancers [110–116]. To date, the following viruses have been identified as being cancer-related: Epstein-Barr virus, Kaposi sarcoma herpes virus, HPV, human T-cell lymphotropic virus, hepatitis B virus, hepatitis C virus, and Merkel cell polyomavirus [25]. A causal effect of HPV on cervical cancer oncogenesis has been confirmed [80], and HPV infection also correlates with the progression of head and neck cancers, esophageal squamous cell carcinoma (ESCC), and bladder cancers [114, 116]. Virome infection also directly causes esophageal squamous cell carcinoma, including HPV, Epstein-Barr virus, and polyoma viruses [114]. Hepatitis B and C viral infections lead to liver cancer [79] and cholangiocarcinoma [112]. Bacteriophages might also be involved in cancer development. Multiple *Streptococcus*-specific bacteriophages and a *Vibrio*-inhabiting bacteriophage have been detected in the gut of patients with CRC compared with controls [117].

### Mechanisms of intratumor microbiome impacts on tumorigenesis

Intratumor microbiome dysbiosis and its clinical significance have been confirmed in clinical samples, but the underlying mechanism remains obscure. The interplay is complex between cancer and the intratumor microbiome, which affects cancer growth and spread by promoting cancer development mainly by increasing mutagenesis, modulating oncogenes or oncogenic pathways, and affecting the immune response.

Many bacteria have evolved to acquire the ability to damage DNA, which could lead to mutational events and eventually contribute to carcinogenesis. Enterobacteriaceae, such as B2 group *E. coli* [118], secrete colibactin and directly induce DNA damage, resulting in colon cancer tumorigenesis [119]. Bacteria with similar functions include *B. fragilis* [34], *H. pylori* [55] and ε- and γ-proteobacteria [119]. Mechanistically, colibactin and cytolethal-distending toxin (CDT) can directly induce DNA damage [119], whereas Bft functions indirectly by producing high levels of reactive oxygen species (ROS) [120]. Chronically high ROS levels can outpace the host DNA repair, and finally results in DNA damage and mutations [38, 88].

**Fig. 2:**
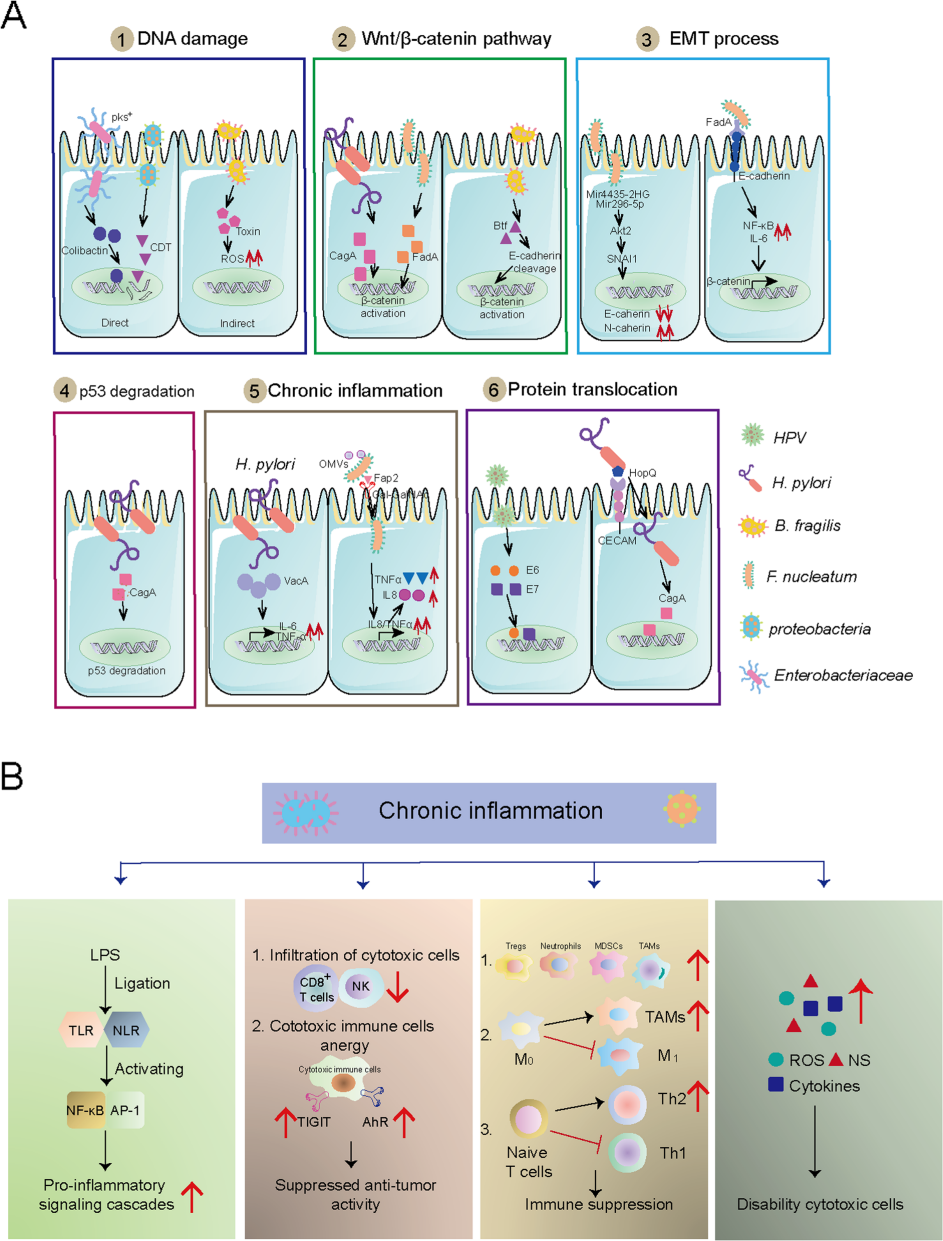
Potential molecular mechanisms by which intratumor microbiota promote carcinogenesis. **A**. Microbiome contributed to the tumorigenesis through inducing DNA damage, Wnt/β-catenin pathway, EMT process, p53 degradation, chronic inflammation and protein translocation. **B**. The chronic inflammation that induced by intratumor microbiota include cancer-associated inflammation, cancer-associated cytokines and ROS/NS production, inhibited cytotoxic immune cells infiltration and function and enhanced immunosuppressive cells infiltration and polarization

Intratumor bacteria are involved in carcinogenesis by producing proteins that participate in host pathways. Among these, the Wnt/β-catenin pathway, an oncogenic signaling pathway in cancer, is altered in many malignancies and is involved in cancer stemness, polarity, and growth [121–123]. This might be because β-catenin activation induces the upregulation of genes involved in cellular proliferation, survival, apoptosis, and migration [121–123]. Several cancer-associated bacteria contribute to activating Wnt/β-catenin signaling. Examples include *H. pylori*, which produces cytotoxin-associated gene A (CagA) protein [52, 124], *F. nucleatum*, which expresses Fn secretes an adhesin A (FadA) [125]and enterotoxigenic *B. fragilis*, which secretes Bft [43]. Mechanistically, CagA can pass into the cytoplasm of host cells and induce gastric cancer by affecting the β-catenin pathway [52, 124], and FadA induces carcinogenesis by activating the β-catenin pathway [125]. Similarly, enterotoxigenic *B. fragilis* produces Bft that stimulates E-cadherin cleavage and subsequently induces β-catenin activation (Fig. 2A) [43].

Intratumor bacteria, such as *F. nucleatum* [63] and *H. pylori* [52] can drive carcinogenesis by inducing degradation of the tumor suppressor gene p53. Other mechanisms explain the tumorigenicity of *H. pylori,* including chronic inflammatory responses and epithelial-mesenchymal transition (EMT) modulation. *Helicobacter pylori* can drive cytotoxicity and chronic inflammation via IL1β, TNFα, and the interferon-gamma (IFN) γ-stimulated Th1-type response [126] or by stimulating TNF-α and IL-6 by secreting vacuolating toxin A (VacA) [55]. *Helicobacter pylori* can modulate host cells through the bacterial protein CagA, which can directly translocate into gastric epithelial cells through the type 4 secretion system (T4SS) [115]. *Fusobacterium nucleatum* is an oncogenic factor in solid tumors, including breast and colon cancer [42, 63, 68]. Mechanistically, *F. nucleatum* stimulates the activation of pro-inflammatory cascades mediated by nuclear factor kappa B (NF-κB) and IL-6, which might facilitate oral squamous cell cancer (OSCC) cell invasion [127]. Outer membrane vesicles (OMVs) produced by *F. nucleatum* can drive chronic inflammation by stimulating colonic epithelial cells to secrete IL-8 and TNF-α [128]. In addition, *F. nucleatum* can induce EMT to drive carcinogenesis [42, 63] (Fig. 2A).

Chronic high-grade inflammation is another mechanism that explains intratumor microbiome-induced oncogenesis [25–27]. Numerous cancer-associated microbiomes induce pro-tumor immune responses. According to data from clinical tissues and animal models, intratumor microbes inhibit innate and adaptive immune systems [15, 129]. The commensal microbiome can stimulate Toll-like receptors (TLRs) via lipopolysaccharide (LPS) that promote proinflammatory signaling cascades to enable a cancer-associated microenvironment. Such bacteria include commensal gram-negative gut bacteria in cholangiocarcinoma [130] and *F. nucleatum* [128]. Activator protein 1 (AP-1) and NF-kB are located downstream of the LPS–TLR axis [131]. Commensal microbes stimulate the production of cancer-associated-cytokines that often have deleterious consequences for tumor progression via activation of the interleukin-23 (IL-23)-IL-17 axis [129], the TNF-α/TNF receptor axis [55, 130], IL-6 family signaling [55, 66, 130], IL-10, IL-8, IL-18, monocyte chemoattractant protein-1 (MCP-1) [125, 132], signal transducer and activator of transcription 3 (STAT3) [55, 133], and the production of ROS [88] and nitrogen species (NS) [38, 120]. Intratumor microbes can directly inhibit anti-tumor immunity by inhibiting cytotoxic immune cell infiltration [42, 61, 71, 131] and blocking their ability to kill tumor cells [124]. Examples include T cell immunoreceptor with Ig and ITIM domains (TIGIT) that is expressed on some T cells and natural killer cells [73] and aryl hydrocarbon receptors (AhRs) expressed on T cells [134]. Commensal microbes recruit abundant inflammatory cells, including tumor-associated macrophages (TAMs), regulatory T cells (Tregs), granulocytes, Vγ6 + Vδ1 + γδ T cells, and myeloid-derived suppressor cells (MDSCs), which results in a pro-inflammatory environment [61, 72, 129, 131, 135, 136] (Fig. 2B).

**Fig. 3:**
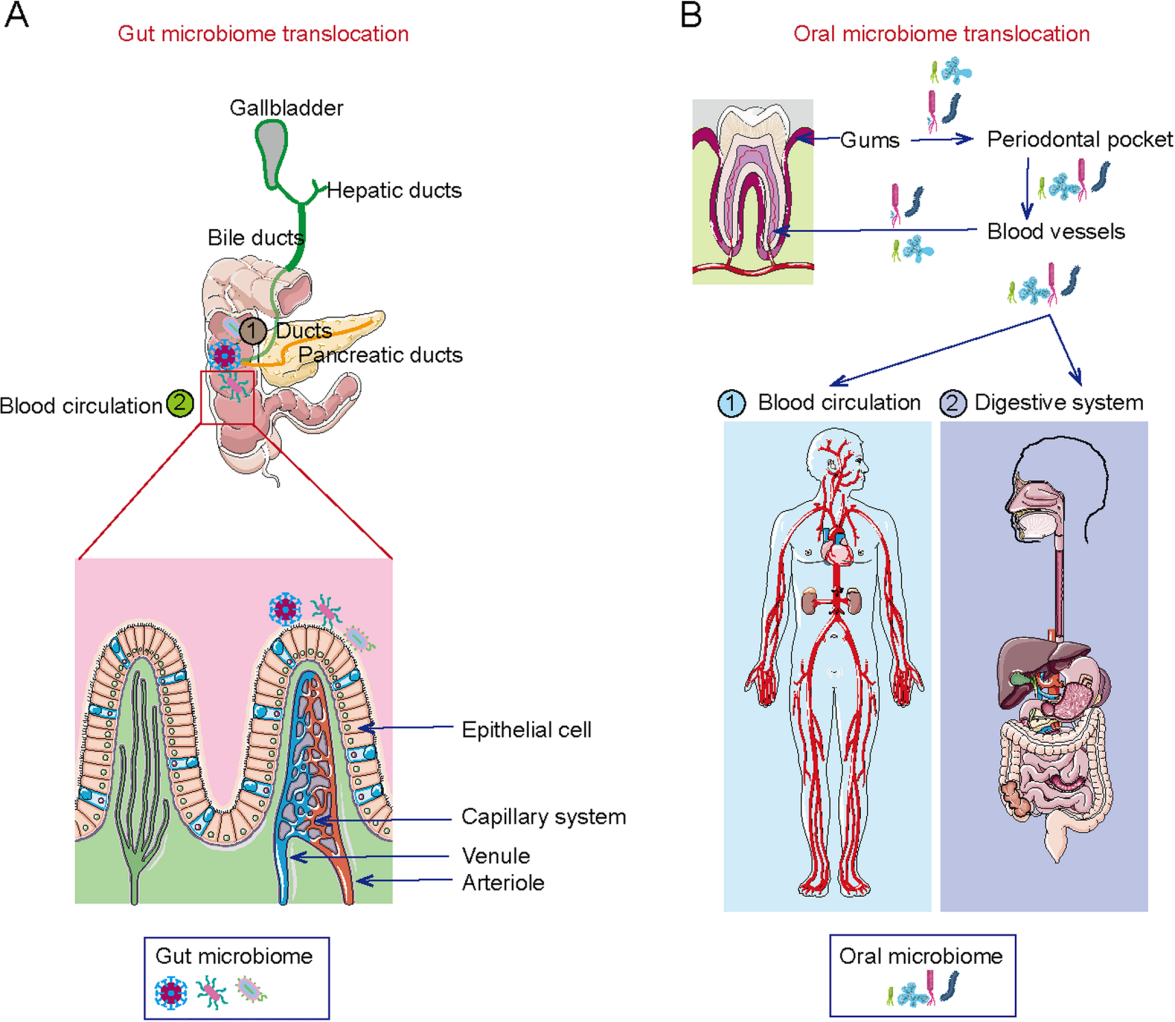
The potential source of intratumor microbiota. **A**. Microbiome may translocate from intestine to the tumor sites, which depends on blood circulation and ducts translocation. **B**. Oral microbiome may be another origin of intratumor microbiota. And blood circulation and digestive system are the main pathways

**Fig. 4:**
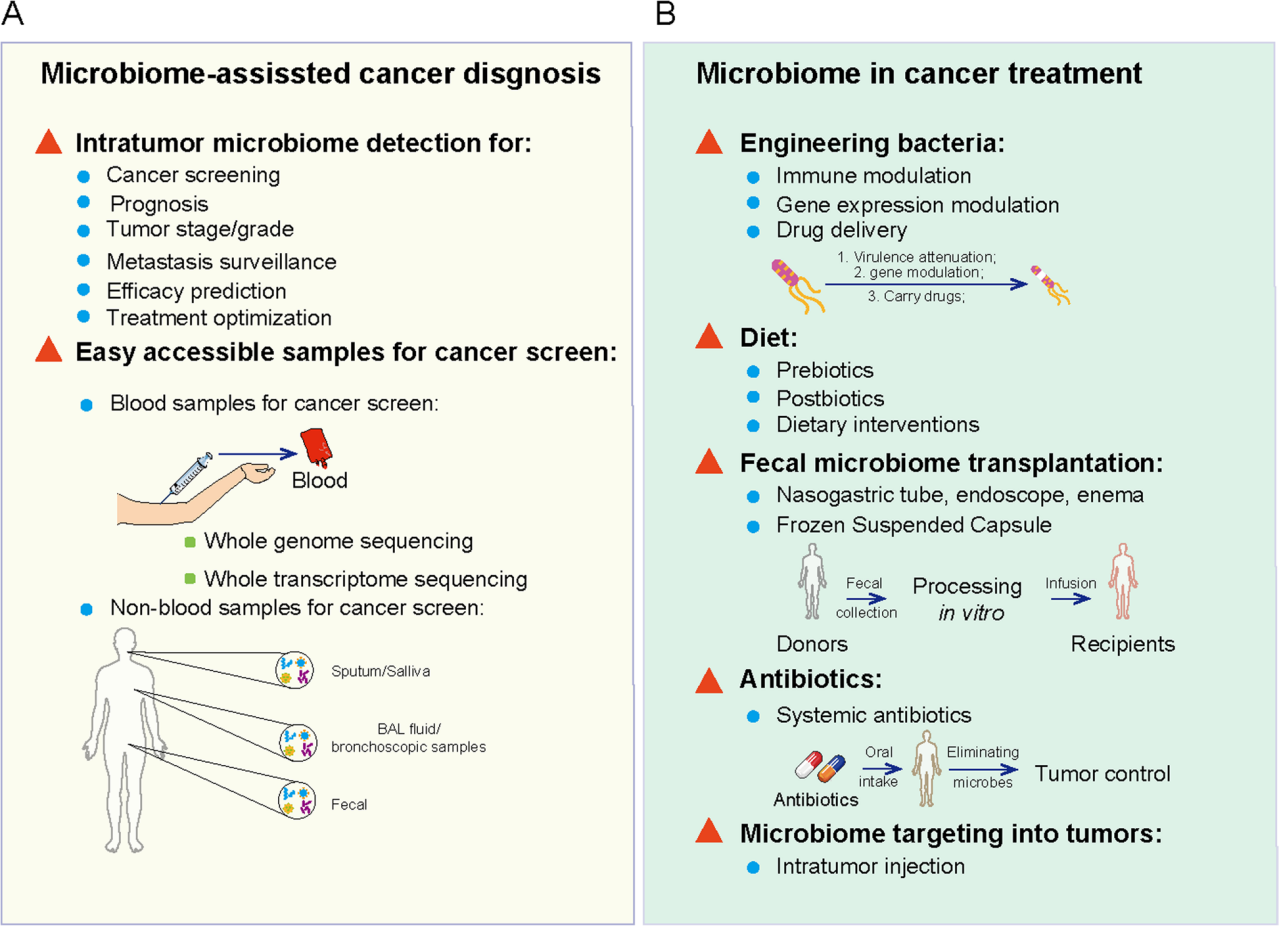
The utilization of intratumor microbiota data in cancer screen and treatment. **A**. Data from clinical samples may facilitate the development of new cancer screen and prognosis, including microbiota patterns from tumor sites and easily accessible samples. **B**. Intratumor microbiota may be applied for cancer treatment, including engineering bacteria, diet modulation, fecal microbiome transplantation, antibiotics and intratumor microbiome injection

The mechanisms of nonbacterial cancer-associated microbial action have not been investigated in detail. Only a few mechanisms might explain non-bacterial tumorigenesis. For example, HPV expresses oncoproteins E6 and E7, which can integrate into the host genome [80, 116], where they trigger the amplification of specific oncogenic genes that induce tumorigenesis in cervical cancer. The putative mechanism of bacteriophage alterations might be through initiating the genetic exchange, which enables ecological adaptations and community networking within hosts, thereby affecting cancer [137]. However, a direct effect of phages on carcinogenesis has not yet been identified. Mycoplasma can induce transformation and tumorigenicity by elevating levels of BMP2, which then increases cell proliferation and migration, and represses apoptosis (Fig. 2A, 3 and 4) [16].

### Microbiota translocation

The commensal microbiome inhabits the mouth, skin, reproductive organs, and gastrointestinal tract in humans [21, 138]. One explanation for the inhabitation of microbiota in tumor sites and the source of this intratumor microbiota is their translocation from the intestine [139].

#### Gut translocation

The gut is the main organ colonized by commensal microorganisms, comprising 3.93 × 10^13^ bacteria [22]. Physically, human and gut microbes inherently coexist symbiotically, and this is partly maintained by spatial separation, explaining the presumed sterility of organs, including the pancreas, breast, kidney, and lung [140]. However, bacterial biomass has been revealed based on the 16S rRNA sequences of bacteria in these organs [15, 61, 141]. Clinical data have revealed that microbiome expression overlaps between tumor tissues and fecal samples, indicating that the intestine is the source of intratumor microbiomes [87, 142]. Despite scant evidence, the microbiome is translocated from the gut into sites in several solid tumors. For example, bacterial profiles are similar in the duodenum and gut, indicating that bacteria can translocate from the gastrointestinal tract into the pancreas [32, 61]. This has been validated in mouse models given fluorescently labeled oral *Enterococcus faecalis*, which confirmed the translocation of bacteria from the gut into the pancreas [61].

The translocation of gram-negative bacteria from the gut, resulting from primary sclerosing cholangitis (PSC) or colitis-induced intestinal barrier dysfunction, finally drives the development of cholangiocarcinoma (CCA) [130]. Translocation of the gut microbiome to the gastrointestinal organ is further validated by the fact that Fusobacteria are maintained in distal liver metastases [70]. Fusobacterium and its associated microbiome comprising *Bacteroides*, *Selenomonas*, and *Prevotella* species overlap in microbiomes in primary colon cancer tissues and paired metastatic liver tumor sites [70]. The microbiome might be translocated from the intestinal tract into other organs via the blood circulation and/or bile, hepatic, and pancreatic ducts [61, 143].

#### Oral translocation

The oral cavity has been regarded as the origin of the intratumoral microbiome [127, 144]. As the entry portal for the gastrointestinal tract, the oral cavity is connected to the respiratory tract, and microbiota residing in the oral cavity can disseminate into the respiratory and gastrointestinal tracts [144]. Therefore, dysbiosis of the oral microbiota, such as periodontal disease, can contribute to the respiratory tract and gastrointestinal cancers, including esophageal cancer [33, 49], head and neck squamous cell carcinoma [145], lung [146], gastric [144], and colorectal [147] types. For example, the esophageal microbiota is similar to the oral microbiota, as it includes Firmicutes, Bacteroides, Actinobacteria, Proteobacteria, Fusobacteria*,* and TM7, indicating the translocation of microbiota from the oral cavity to the esophagus [44]. Furthermore, the results of organisms cultured from aspirates obtained from patients with esophageal cancer and healthy persons during esophagoscopy revealed that *Haemophilus influenzae*, *Moraxella catarrhalis*, *and Streptococcus spp* in the esophagus overlap with oral microbiota [33, 114, 148–150]. Additionally, oral *Prevotella intermedia*, *Tannerella forsythia*, and *Treponema denticola* possess peptidyl arginine deaminase (PAD) that induces P53 mutations in pancreatic cancer [151].

Periodontal disease, characterized by dysbiosis in oral microbiota, is associated with the risk of genitourinary cancers, including bladder and prostate cancers [49, 141, 144–147, 152, 153]. Likewise, a comparison of microbiota in chronic prostatitis and BPH revealed overlapping *P. gingivalis*, *Treponema denticola,* and *E. coli* in the oral cavity and prostatic tumor sites [118]*.* The detection of oral microbiota in genitourinary organs suggests a potential role of oral microbiota in the initiation and progression of genitourinary cancers through the oral-genitourinary axis. Although epidemiological studies have identified interplay between periodontitis and various types of cancers and a close correlation between them, the causal impact of oral microbiota on genitourinary cancers remains mechanistically speculative. This is because the periodontium where the oral microbiota originally resides is located far from the genitourinary organs. The migration of oral microbiota from the oral cavity to distant organs has been investigated. The translocation of oral bacteria to the respiratory tract and gastrointestinal organs might be via the bloodstream and the digestive tract [154]. The proposed concept of oral bacterial translocation via the bloodstream is based on the anatomical structure and the proximity of the periodontal pockets to the bloodstream.

### Potential clinical application of intratumor microbiota

#### Cancer screening and diagnostics

The microbiota species significantly differ between tumor and healthy tissues, and some bacteria are causally associated with cancer development [155]. This indicates that the intratumor microbiota could function as a biomarker for cancer screening [139] [40]. Examples include intratumor microbiome-derived personalized data that can distinguish patients with esophageal [47], pancreatic [35], lung [93] and oral [64] cancers from healthy persons. In addition to cancer diagnosis, microbiome signatures also differ among tumor stages, tumor grades, cancer scores [60, 81], gene mutations (such as estrogen, progesterone receptors, and Her2) [156] or regions [157]. Furthermore, the unique intratumor microbiome signature correlates with distant tumor metastasis [42, 63] and responses to chemotherapy [50, 89]. Profiling the intratumor microbiome might offer potential for tumor prognostic evaluation. Indeed, intratumor microbiome diversity is higher and *Pseudoxanthomonas, Streptomyces, Saccharopolyspora, and B. clausii* are upregulated in patients who have survived PDA over the long-term [62]. Similarly, *F. nucleatum* signatures have been identified in esophageal cancer and the correlation with prognosis [46].

As understanding of the intratumor microbiome effects on cancer pathogenesis deepens, the application of these profiles to precision oncology is highly attractive. However, obtaining organ biopsies from healthy humans is unethical, and obtaining samples for cancer screening is difficult. Alternatives to inaccessible and unethical biopsies in healthy humans include saliva, sputum, bronchoscopy samples, and BALF [37, 39, 93] to screen for lung cancer, the tongue microbiome to screen for pancreatic cancer [158], and the oral microbiome to screen for esophageal cancer [159]. The existence of microbiome in blood is verified in colorectal and breast cancer patients according to the data from The Cancer Genome Atlas (TCGA) [160]. The blood microbiome is unique and can distinguish cancer based on whole-genome and -transcriptome sequencing data for 33 types of cancer from treatment-naive patients (18,116 samples) in TCGA [139].

Systematic characterization of the microbiota provides an opportunity to explore new methods for non-human microorganism-derived molecules in cancer diagnosis. Moreover, new strategies involving easily accessible samples are needed to screen for cancer without invasive testing. However, many challenges must be tackled for intratumor microbiome-based clinical diagnoses, such as inaccessible tumor tissues, relatively low microbiome biomass, and easy contamination. In addition, the specificity, prevalence, and stability of the intratumor microbiome during cancer treatment must be addressed before clinical deployment.

### Intratumor microbiome modulation and clinical efficacy

Cancers have a dismal prognosis, and outcomes have improved little with modern [161–164] and traditional [59, 165, 166] therapeutic strategies. Apart from tumor cell-intrinsic mechanisms, discovering the intratumor microbiome and elucidating host-microbe interactions present opportunities for intervention [1, 2, 167]. Increasing evidence suggest that microbiota modulation is currently recognized as a novel and important adjunct to enhance anticancer therapies [27, 168, 169]. Efforts to use microbiota for cancer treatment during the twentieth century [20] were unsuccessful. The intratumor microbiome has recently been extensively investigated [15] and tumor-targeting bacteria such as *Salmonella typhimurium* strain VNP20009 [170], *Listeria monocytogenes* [171] and *Listeria* spp. [172] can selectively eliminate tumor cells. Owing to their ability to selectively colonize in tumors or tumor-driven lymph nodes to inhibit tumor growth, preclinical studies have evaluated treatment efficiency in mouse models [170, 172]. The promising anti-tumor responses in preclinical studies have led to the selection of several bacterial strains for evaluation in patients with tumors [173, 174]. However, the clinical outcomes are unsatisfactory; only objective responses or minor and transient side effects have been identified in clinical trials [173, 175]. The discrepancies between outcomes in preclinical researches and clinical trials might be explained by differences in tumor structures and growth rates, which could change bacterial penetration, proliferation, and clearance within tumors, as well as in peripheral circulation.

Also, live bacteria could be attenuated and further reprogrammed to produce and deliver anticancer agents based on clinical requirements [176, 177]. Tumor-targeting bacteria offer several advantages as delivery vectors, including improved penetrability of tumor sites, maximized activities of chemotherapeutic agents, and reduced systemic toxicity. Regulating bacterial gene expression might further modulate the accumulation of anti-tumor payloads at tumor sites and control continuous drug delivery [132]. Several strategies have been developed to selectively deliver tumor-targeting bacteria, including cytokines, chemotherapeutic agents, prodrug-converting enzymes, small interfering RNAs (siRNAs), and immunomodulators [95]. These have enhanced anti-tumor responses and reduced nonspecific side effects in tumor models [178–180]. Considering the role of the intratumor microbiota in modulating host immunity [102, 181], it could probably influence responses to and toxicity of various types of cancer therapy. Nevertheless, direct control of intratumor microbiome modulation is still a long way off. Obstacles need to be overcome, such as controlling microbiome toxicity, inaccessible microbiomes in tumor sites, potential side effects and the accuracy of microbiome biomass delivery into tumor sites.

Since the oral cavity and intestine are recognized as the primary sources of the intratumor microbiome, modulation of the microbiome in the gut might reshape that in tumors and affect cancer therapies. Cross-talk between the gut and intratumor microbiomes has been identified in pancreatic cancer [61, 62, 96]. Thus, gut microbial modulation via antibiotics, diet, and fecal microbiota transplantation (FMT) might have the potential as a powerful immunotherapeutic modality. Antimicrobial therapy in cancer is limited to address or prevent known cancer-associated microbiomes, such as HPV, *H. pylori*, HBV/HCV, Epstein-Barr virus, and polyoma virus-induced cancers [79, 80, 112, 114, 116]. Nevertheless, systemic antibiotics are not always beneficial, as they can weaken the immune checkpoint blockade (ICB) and result in a poor prognosis [182, 183]. Prebiotics, postbiotics, and dietary interventions are also regarded as promising strategies to improve anti-tumor immunity and therapy responses to cancers in both mouse models [17, 184] and clinical trials (NCT03870607, NCT03950635) [136, 184]. However, collecting dietary data has restrained elucidation of the causal mechanisms underlying this strategy. Instead, metabolomic data that can reveal dietary intake and concomitant small-molecule effectors might serve as a substitute for mechanistic exploration. In addition, the gut microbiota has been modulated using FMT to enhance ICB efficacy. The results of mouse models [21, 142] and a clinical trial (NCT03353402) have been promising [23, 30, 31, 136]. Many factors complicate the use of FMT in clinical cancer treatment, such as the complexity of monoclonal bacterial strains, multiplexed consortia, antibiotic preconditioning, administration routes, the modulation frequency, and dietary recommendations. However, the long-term efficacy and stability of FMT in cancer treatment have not been evaluated.

## Concluding remarks

The expression of the intratumor microbiome in patients with cancer has gradually been revealed due to technological developments [15]. Although many intratumor microbiome dysbiosis in solid cancers contribute to oncogenesis, progression, and drug resistance, the direct causal roles and underlying mechanisms of the intratumor microbiome remain ambiguous. Gaining sufficient insight into modes of action through which the microbiome might function as a biotherapeutic agent is important for patient prediction and the successful, rational development of microbiome-modulating therapies to enhance clinical treatment effects. Efforts have been targeted towards the application of gut microbiota to modulation-based cancer therapies because of cross-talk between intratumor and gut microbiota. Modulating the gut microbiome to treat cancer has been attempted, but causal mechanisms of adjuvants are difficult to reveal in complex environments. Identifying monoclonal bacterial strains that are beneficial to the anti-tumor response is imperative. Tumor-specific microbiomes have been confirmed [15]. Therefore, precisely characterizing the components of the tumor microbiome would provide valuable insights that might facilitate the development of tumor-specific treatments without severe side effects.

## Data Availability

Data sharing not applicable to this article as no data-sets were generated or analyzed during the current study.

## Abbreviations

TME: Tumor microenvironment
NGS: Next-generation sequencing
CRC: Colorectal cancer
ECCA: Extrahepatic cholangiocarcinoma
HPV: Human papillomavirus
GBM: Glioblastoma multiforme
BALF: Bronchoalveolar lavage fluid
BMP2: Bone morphogenetic protein 2
BPH-1: Benign prostate hyperplasia
PDA: Pancreatic ductal adenocarcinoma
MBL: Mannose-binding lectin
TNF-α: Tumor necrosis factor-alpha
IL-18: Interleukin (IL) 18
ESCC: Esophageal squamous cell carcinoma
CDT: Cytolethal-distending toxin
ROS: Reactive oxygen species
CagA: Cytotoxin-associated gene A
FadA: Fn secretes an adhesin A
IFN γ: Interferon-gamma (IFN) γ
VacA: Vacuolating toxin A
T4SS: The type 4 secretion system
NF-κB: Nuclear factor kappa B
OMVs: Outer membrane vesicles
TLRs: Toll-like receptors
LPS: Lipopolysaccharide
AP-1: Activator protein 1
MCP-1: Monocyte chemoattractant protein-1
NS: Nitrogen species
AhRs: Aryl hydrocarbon receptors
TAMs: Tumor-associated macrophages
MDSCs: Myeloid-derived suppressor cells
PSC: Primary sclerosing cholangitis
CCA: Cholangiocarcinoma
PAD: Peptidyl arginine deaminase
siRNAs: Interfering RNAs
FMT: Fecal microbiota transplantation
ICB: Immune checkpoint blockade
EMT: Epithelial-mesenchymal transition
OSCC: Oral squamous cell cancer
